# *Clostridium butyricum* ameliorates indomethacin-induced enteropathy by promoting MUC2 secretion via suppressing the Notch pathway

**DOI:** 10.3389/fmicb.2025.1509876

**Published:** 2025-03-19

**Authors:** Lanping Zhu, Yang Luo, Yaxin Liu, Siyuan Sun, Junjie Yuan, Lijun Zhang, Weilong Zhong, Shuang Ma, Zihan Yu, Jinjie Zhou, Xin Chen, Jingwen Zhao

**Affiliations:** Department of Gastroenterology and Hepatology, Tianjin Medical University General Hospital, Tianjin, China

**Keywords:** small intestinal damage, non-steroidal anti-inflammatory drugs, *Clostridium butyricum*, Notch pathway, mucin MUC2

## Abstract

Nonsteroidal anti-inflammatory drug (NSAID) enteropathy is a serious clinical complication with no effective treatments available. Modulating the intestinal microbiota through dietary and nutritional targets is a promising strategy for preventing NSAID enteropathy. This study aimed to investigate the protective effect and underlying mechanisms of the probiotic *Clostridium butyricum* (CB) on indomethacin (IND)-induced enteropathy. C57BL/6J mice received CB treatment for 14 days along with concurrent IND gavage for the final 7 days. Caco2 cells were stimulated with IND to evaluate the effect of CB supernatant (CBS) on the intestinal barrier function, and LS174T cells were used to validate the modulatory action of CBS on the Notch signaling pathway. Our findings revealed that CB treatment prevented anorexia and weight loss, reduced the severity of enteropathy, and decreased the inflammatory response of the small intestine. CB also increased the expression of tight junction proteins and reduced permeability in mice and Caco2 cells. Additionally, CB suppressed apoptosis and promoted proliferation in the small intestine. Further research found that CB increased the number of goblet cells and MUC2 secretion. Mechanistically, CB may promote MUC2 secretion by suppressing the Notch signaling pathway, consistent with the results of intervention in LS174T cells with CBS. In conclusion, CB might prevent NSAID enteropathy by increasing MUC2 secretion through the inhibition of the Notch pathway. Our study identified the potential efficacy of CB as a preventive strategy against NSAID enteropathy and showed promising prospects for CB as a food supplement.

## 1 Introduction

Nonsteroidal anti-inflammatory drugs (NSAIDs) are widely used worldwide as potent antipyretic, analgesic, anti-inflammatory, and antiplatelet agents. However, the use of NSAIDs is often associated with adverse effects on the gastrointestinal (GI) tract, including severe complications such as bleeding, perforation, and ulcers (Bjarnason et al., 2018). Furthermore, capsule endoscopy and balloon-assisted endoscopy have revealed that NSAIDs can also cause damage to the small intestine. The small intestine injury caused by NSAIDs manifests as erythema, erosion, ulcers, bleeding, and diaphragm-like strictures (Watanabe et al., 2020). The precise cause of this injury, which involves multiple factors including elevated intestinal permeability, deficiency of prostaglandins, and the presence of bile acids, but is still not completely understood (Bjarnason et al., 2018). Unfortunately, timely detection of this type of damage is challenging. Therefore, effective strategies for preventing NSAID-induced damage should be developed.

Recent studies have shown that dysbiosis of the microbial community in the small intestine plays a role in the development of NSAID-induced enteropathy, which is distinct from the damage seen in the upper gastrointestinal tract (Wallace et al., 2011; Nadatani et al., 2019). Some clinical studies have suggested that probiotics may provide protection against NSAID enteropathy, although the exact mechanism of action remains unclear (Suzuki et al., 2017; Mortensen et al., 2019; Chen et al., 2022). Therefore, modulating the small intestinal microbiota in a beneficial way may be a promising approach for the treatment of NSAID enteropathy. *Clostridium butyricum* (CB) is a probiotic that has been demonstrated to exhibit protective or ameliorative effects against various diseases, such as irritable bowel syndrome (IBS), inflammatory bowel disease (IBD), metabolic disorders, and colorectal cancer (Yasueda et al., 2016; Sun et al., 2018; Perraudeau et al., 2020; Zhang K. et al., 2023). However, there is currently a lack of studies investigating the use of CB specifically for the prevention and treatment of NSAID enteropathy.

The mucus layer is a physical barrier in the gastrointestinal tract. It protects the mucosa from invasion by bacteria and harmful agents (Kang et al., 2022). Mucin2 (MUC2), which is secreted by goblet cells, is a highly glycosylated protein and is a key mucin protein involved in mucosal protection (Holmén Larsson et al., 2013). Lack of MUC2 was found to cause spontaneous colitis (Lu et al., 2011). The Notch signaling pathway is involved in the regulation of MUC2 (Jiang et al., 2022; Yan et al., 2022). Activation of the Notch pathway leads to the induction of HES-1, a transcriptional repressor that suppresses the expression of MATH-1 (Wang et al., 2015). Inhibition of the Notch signaling pathway can result in the upregulation of MATH-1, which binds to the E-boxes of the MUC2 promoter and promotes the transcription and expression of MUC2 (Park et al., 2006; Vooijs et al., 2007). Therefore, suppression of the Notch signaling pathway may enhance the secretion of MUC2, thereby contributing to mucosal protection.

In this study, we conducted a comprehensive investigation to assess the effect of CB on the small intestinal mucus barrier. We aimed to understand how CB regulates the Notch signaling pathway and promotes MUC2 secretion. These findings hold promise for the development of novel preventive approaches utilizing probiotics to protect against NSAID-induced intestinal injury.

## 2 Materials and methods

### 2.1 Animals

The C57BL/6 mice (6–8 weeks old; 19–21 g, SPF) were obtained from Beijing Huafu Kang Bioscience Co., Ltd. The mice were kept in an environment with a 12 h light/dark cycle and a temperature of 24–27°C, with free access to food and water. The animal study protocol was approved by the Ethics Committee of the Institute of Radiation Medicine, Chinese Academy of Medical Sciences (IRM-DWLL-2023128).

### 2.2 Bacterial culture and supernatant preparation

*Clostridium butyricum* (CGMCC313-1) was cultured in reinforced clostridial medium (Beosen, China) under anaerobic conditions at a constant temperature of 37°C for 48 h. Then, it was incubated to reach the logarithmic phase, and the optical density was determined to be 0.5 at A600. CB was harvested via centrifugation and resuspended in sterile PBS for immediate use. The amount of CB was estimated by dilution method of plate counting, and 10^8^ colony forming units (CFUs)/mL was obtained. The culture medium was centrifuged at 3000 g for 20 min at 4°C and filtered through a 0.2 mm pore-size filter to yield the CB supernatant (CBS).

### 2.3 Animal experimental design

Animals were randomly divided into three groups (*n* = 6 mice/group). (1) In the control (CON) group, the mice were gavaged with 200 μL of phosphate buffered salt (PBS; Sigma, United States) per day for 14 days and 0.5% carboxymethylcellulose (CMC; Thermo, United States) per day for the last 7 days. (2) In the indomethacin (IND) group, the mice were gavaged with 200 μL of PBS for 14 days, followed by oral IND (Sigma, United States; 5 mg/kg; suspended in 0.5% CMC) for the last 7 days. In the CB + IND group, the mice were gavaged with 200 μL of CB (1 × 10^8^ CFU, suspended in PBS) for 14 days and IND for the last 7 days. A model of NSAID enteropathy was established on the basis of previous studies (Whitfield-Cargile et al., 2016). The dose of CB was based on previous literature (Zhao et al., 2019). Prior to the start of the experiments, all animals were acclimated for a week. During the experiment, body weight and food intake were recorded daily. On day 15, the mice were euthanized, and blood was collected from the retro-orbital vein. The small intestine was removed, and the relative length between the pyloric valve and the ileocecal valve was measured. The last third of the small intestine was collected and cut in half. The distal small intestine was fixed in 4% formaldehyde, and the rest of the small intestine was stored at −80°C to evaluate the mRNA expression in each group.

### 2.4 Histological analysis and tissue damage index scores

The small intestine tissue was fixed in 4% paraformaldehyde, dehydrated, and paraffin-embedded. Then, it was cut into 5 μm-thick paraffin sections, dehydrated with ethanol and xylene, and stained with hematoxylin and eosin (H&E). All paraffin sections were observed with optical microscopy, and Chiu’s scale was used to evaluate the level of small intestinal damage (Chiu et al., 1970).

### 2.5 Immunofluorescence staining

To investigate the neutrophil infiltration and the expression of Notch-1 receptor in the small intestine, paraffin sections were dewaxed, hydrated, and occluded with 10% serum for 30 min at ambient conditions. Then they were incubated with a primary antibody anti-Ly6G (1:500, Signalway Antibody, #62028, United States) and antibody anti-Notch-1 (1:50, Signalway Antibody, #30991, United States) respectively overnight at 4°C. Subsequently, the sections were incubated with an Alexa Fluor 488 conjugated goat anti-rabbit IgG antibody (1:500, Invitrogen, A-11008, United States) for 1 h at room temperature. Finally, each section was stained with a gold antifade agent containing DAPI working solution (1:500; Sigma, United States) for 5 min and analyzed through a Leica fluorescence microscope (Leica, Germany).

To detect the alterations of key regulatory factors in Notch signaling pathway, after paraffin sections were ready, they were first co-incubated with MATH-1 antibody (1:200; Bioss, bs-3522R, China) overnight at 4°C. Then, each section was incubated with CY3 working solution (1:200; appeared red; Solarbio, China) for 10 min at ambient conditions. Subsequently, it was incubated with 10% serum at room temperature for 30 min and co-incubated with antibody HES-1 (1:200, Bioss, bsm-52568R, China) at 4°C overnight. Each section was then incubated for 10 min at room temperature with FITC working solution (1:300; appeared green; Solarbio, China). Finally, each section was stained with an appropriate amount of DAPI working solution (1:500; Sigma, United States) for 5 min, and all the sections were sealed with a fluorescent sealer and observed under the fluorescence microscope.

### 2.6 Immunohistochemical assay

The expression of intestinal barrier proteins (ZO-1, occludin, and claudin-1), Ki67, cleaved caspase-3, MUC2, and MUC5AC was evaluated by IHC. Paraffin sections were deparaffinized in xylene and then rehydrated. The rehydrated tissue sections were microwaved for antigen extraction and incubated with primary antibodies ZO-1 (1:500, Abcam, ab276131, UK), occludin (1:200, Abcam, ab216327, UK), claudin-1 (1:800, PTG, 28674-1-AP, United States), Ki67 (1:400; Thermo, MA5-14520, United States), cleaved caspase-3 (1:200; Thermo, PA5-114687, United States), MUC2 (1:500; Signalway Antibody, #53898, United States) and MUC5AC (1:200, Novus Biologicals, NBP2-15196, United States) at 4°C overnight. After being washed three times by PBS, they were further incubated with suitable horseradish peroxidase-labeled secondary antibody. After images were collected, quantitative IHC analysis was performed with ImageJ.

### 2.7 Periodic acid–Schiff staining

For measurement of the number of goblet cells, paraffin sections were subjected to PAS staining in accordance with the instructions of the glycogen PAS staining kit (Solarbio, China). The goblet cells were observed on 100 successive small intestinal villi with the light microscope.

### 2.8 ELISA

Small intestinal permeability was evaluated by measuring serum D-lactate (D-LAC) levels with D-LAC assay kit (Sbjbio, China) and enzymatic spectrophotometry method. As directed by the CALP assay kit (Sbjbio, China), the inflammation of the small intestine was evaluated by measuring the calprotectin levels in the contents of the small intestine via enzymatic spectrophotometry method.

### 2.9 Cell culture

Caco2 and LS174T cells were purchased from the Chinese Academy of Sciences Cell Bank (Shanghai, China). Differentiated Caco2 exhibits a phenotype similar to human small intestinal epithelium both structurally and functionally and therefore is used widely as a cell model of the NSAID enteropathy (Omatsu et al., 2009; Fukui et al., 2012; Kim et al., 2019). The Caco2 cells were cultured in modified Eagle’s medium (MEM; Solarbio, China) with 15% fetal bovine serum (FBS; Gibco, United States), 0.5% penicillin–streptomycin (Solarbio, China), and 0.5% non-essential amino acids (Solarbio, China). We investigated the effects of CBS on expression of MUC2 using the LS174T cell line, which mimics goblet cell and expresses mucins (van Klinken et al., 1996; Kim et al., 2019). The LS174T cells were cultured in Dulbecco’s MEM (Solarbio, China) with 15% FBS, 0.5% penicillin–streptomycin, and 0.5% non-essential amino acids. The cells were cultured in a humidified environment containing 5% carbon dioxide at 37°C.

### 2.10 Cell viability assay

Cell Counting Kit-8 (CCK-8; Beyotime, China) was used to detect the effect of IND or CBS on the viability of Caco2 cells. The Caco2 cells (5 × 10^3^) were inoculated in 96-well plates. When the cell density grew to 60–70% in each well, the cells were exposed to different concentrations of IND [0, 100, 200, 300, 400, and 500 μM; dissolved in dimethyl sulfoxide (DMSO; Sigma, United States)] for 24 h. Subsequently, the Caco2 cells were treated in advance with different concentrations of CBS (0, 1:150, 1:100, 1:50, 1:25, and 1:10) for 8 h and incubated with IND for 24 h. Then, 20 μL/well of CCK-8 solution was added to the cell culture medium and incubated at 37°C for 4 h. The optical density (OD) was measured at 450 nm with an enzyme marker. The LS174T cells were also seeded at a density of 5 × 10^3^ per well in 96-well plates. They were exposed to different concentrations of CBS (1, 2, 5, 7, and 10%) for 48 h, and the OD was determined at 450 nm after adding CCK-8 in the same manner as above.

### 2.11 Transepithelial electrical resistance measurement

Transepithelial electrical resistance (TEER) was used to evaluate the integrity of the cell monolayer model. Caco2 cells (5 × 10^4^) were inoculated in Transwell chambers (Labselect, China). The model is generally considered to be successfully established when its TEER value is ≥500 Ω·cm (Yang et al., 2022). The Caco2 cells were treated in advance with CBS for 8 h and incubated with IND for 24 h. The TEER value was measured at 0, 3, 6, 9, 12, 15, 18, 21, and 24 h with the Millicell ERS-2 instrument (Millipore, United States) by using the following formula: TEER (Ω·cm^2^) = (total resistance − empty resistance) (Ω) × area (cm^2^).

### 2.12 Measurement of Notch signaling in LS174T cells

LS174T cells were treated with different concentrations of CBS for 48 h. Then, the mRNA and protein expression levels of the corresponding Notch signals were determined by quantitative real-time polymerase chain reaction (qRT-PCR) and Western blotting.

Valproic acid (VPA; Glpbio, China; dissolved in DMSO) can activate Notch signaling (Adler et al., 2008). LS174T cells were treated with 5% CBS in the presence or absence of 50 or 100 μM VPA for 48 h. The corresponding mRNA and protein expression levels of Notch signaling were determined by qRT-PCR and Western blotting.

### 2.13 Quantitative real-time polymerase chain reaction

In accordance with the reagent instructions, total RNA was extracted from mouse small intestine tissue or cells by using TRIzol reagent (Vazyme, China). cDNA was synthesized from the extracted RNA by using the HiScript III RT SuperMix for qPCR kit (Vazyme; Nanjing, China). Subsequently, qRT-PCR was performed using ChamQ Universal SYBR qRT-PCR Master Mix (Vazyme, China). The levels of the target genes were normalized to those of the housekeeping gene GAPDH by using 2^−ΔΔCt^ method to calculate the relative mRNA levels. All the primer sequences are listed in Supplementary Table S1.

### 2.14 Western blotting

LS174T cells were lysed in RIPA buffer (Solarbio, China) containing protease inhibitor, and the total proteins were extracted. The protein concentrations were evaluated using the BCA protein assay kit (Solarbio, China). The proteins from each sample were separated by sodium dodecyl sulfate-polyacrylamide gel electrophoresis and transferred to blank polyvinylidene fluoride (PVDF) membranes (Solarbio, China) at 90 V. The PVDF membranes were blocked with 10% milk at room temperature for 1 h. The bands were incubated with anti-MATH-1 (1:1000; Bioss, bsm-61775R, China), anti-HES-1 (1:1000, Bioss, bsm-52568R, China), anti-MUC2 (1:1000; Signalway Antibody, #53898, United States), anti-Notch-1 (1:1000, Signalway Antibody, #30991, United States), and anti-β-tubulin (1:1000; Signalway Antibody, #44032, United States) overnight at 4°C. Subsequently, the bands were incubated with goat anti-mouse or anti-rabbit IgG secondary anti-bodies for 1 h at room temperature. Finally, they were observed by enhanced chemiluminescence, and the densitometry of the immunoblots was quantified by ImageJ software.

### 2.15 Statistics

GraphPad Prism version 7.00 software was used to perform statistical analysis and create the graphs. Data were shown as mean ± standard deviation. Data normality was tested by the Shapiro–Wilk test. One-way ANOVA was used for comparison of more than three groups. The Kruskal-Wallis test was performed for analyzing data with skewed distribution. *p* value < 0.05 was regarded as a statistically significant difference.

## 3 Results

### 3.1 CB ameliorated clinical signs in IND-induced enteropathy mice

**Figure 1:**
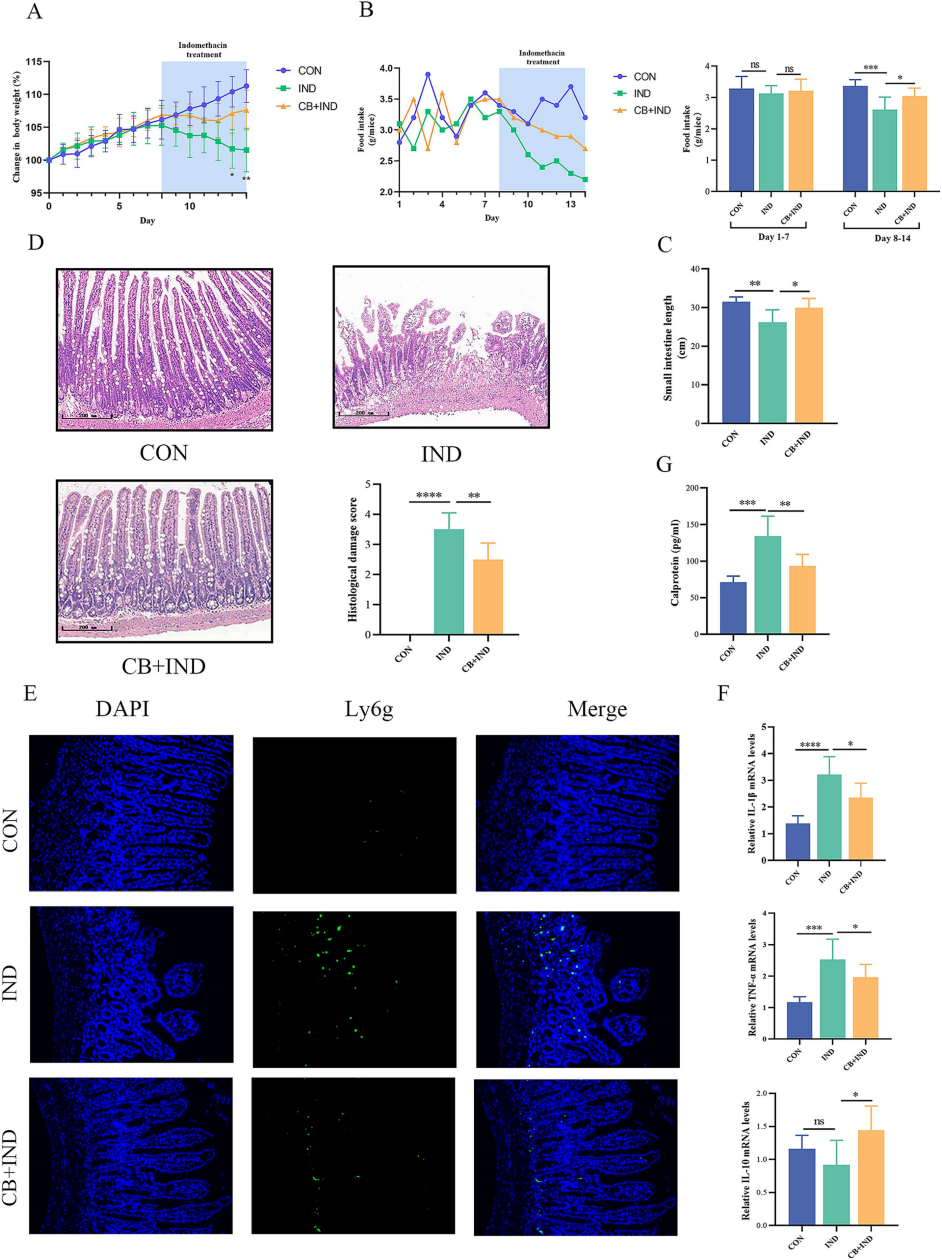
*Clostridium butyricum* (CB) ameliorated indomethacin (IND)-induced enteropathy and reduced the inflammation response in mice. **(A)** Body weight changes of mice. **(B)** Food intake changes of each group. **(C)** Length of small intestine of mice. **(D)** Representative H&E staining of each group (scale bar = 200 μm) and histological damage score. *n* = 6. **(E)** Immunofluorescence staining for lymphocyte antigen 6 complex, locus G (Ly6G, scale bar = 100 μm, *n* = 3). **(F)** Relative mRNA expression of IL-1β, TNF-α, and IL-10, as analyzed via qRT-PCR (*n* = 6). **(G)** Level of fecal calprotectin of mice, as measured by ELISA (*n* = 6). Data were presented as mean ± standard deviation. ns, no significance; *p < 0.05, **p < 0.01, ***p < 0.001, and ****p < 0.0001.

IND-induced enteropathy causes intestinal dysfunction, which leads to reduced food intake and weight loss in mice. The effect of oral administration of CB was assessed in IND-induced enteropathy model mice. During the first 7 days after gavage, no significant difference was found in the body weight and food intake among the groups. From day 8 to day 14, compared with the CON group, the body weight and food intake of the mice treated with IND decreased over time. In contrast, the IND-induced weight loss and decreased food intake were effectively alleviated in the CB + IND group (Figures 1A,B).

The shortening of the small intestine is positively associated with inflammation and edema caused by NSAID enteropathy. The small intestinal length in the IND group was significantly shortened compared with that in the CON group (26.17 ± 3.26 cm versus 31.46 ± 1.28 cm, *p* < 0.01), and CB treatment prevented IND-induced shortening of the small intestine in the CB + IND group (29.91 ± 2.43 cm, *p* < 0.05, Figure 1C).

The pathological appearance of the small intestine was evaluated through microscopic observations. The severity of injury was assessed by Chiu’s score from 0 to 5. The IND group had varying levels of mucosal thinning, ulceration, and villi loss. By contrast, CB administration greatly improved the extent of IND-induced pathological damage to the small intestinal mucosa. The small intestinal mucosa appeared intact, with mild edema of the villi, no obvious necrosis or detachment of the villi, and a lower pathological damage score in the CB + IND group (*p* < 0.01) (Figure 1D).

### 3.2 CB reduced the inflammatory response

NSAID enteropathy is typically characterized by inflammatory responses and excessive activation of inflammation, which can worsen intestinal mucosal damage. Neutrophil infiltration plays a vital role in NSAID enteropathy, and the lymphocyte antigen 6 complex, locus G (Ly6G), is a specific marker of neutrophils, which could be used as a marker of neutrophil infiltration (Simeoli et al., 2017). In the IND group, a large number of Ly6g-positive cells was found in the region of the damaged small intestinal mucosa. These cells infiltrated into the lamina propria, whereas in the CB + IND group, neutrophil infiltration was reduced (Figure 1E).

TNF-α is a pro-inflammatory cytokine that can be produced by activated monocytes/macrophages, and it is capable of triggering an inflammatory cascade by promoting the expression of cytokines (e.g., IL-1β), thus playing an essential role in NSAID enteropathy (Watanabe et al., 2020). In the present study, TNF-α and IL-1β mRNA expression significantly increased in the IND group compared to the CON group (*p* < 0.001 and *p* < 0.0001), but they were markedly reduced by CB treatment (*p* < 0.05 and *p* < 0.05, Figure 1F). Meanwhile, IL-10 can act as an anti-inflammatory cytokine to suppress intestinal inflammation. In this work, there was no statistically significant difference in IL-10 expression between the CON and IND groups, but CB treatment did significantly increase IL-10 expression compared to the IND group (*p* < 0.05, Figure 1F).

Calprotectin, a Ca^2+^-binding protein from neutrophils, is strongly correlated with inflammation levels and is a non-invasive biomarker of intestinal inflammation (Ayling and Kok, 2018). The IND group had a significantly higher fecal calprotectin levels compared to the CON group (134.07 ± 24.41 pg./mL versus 71.25 ± 8.29 pg./mL, *p* < 0.001). In contrast, the fecal calprotectin levels decreased in the CB + IND group compared to the IND group (93.63 ± 15.67 pg./mL, *p* < 0.01, Figure 1G).

### 3.3 CB protected intestinal barrier function

**Figure 2:**
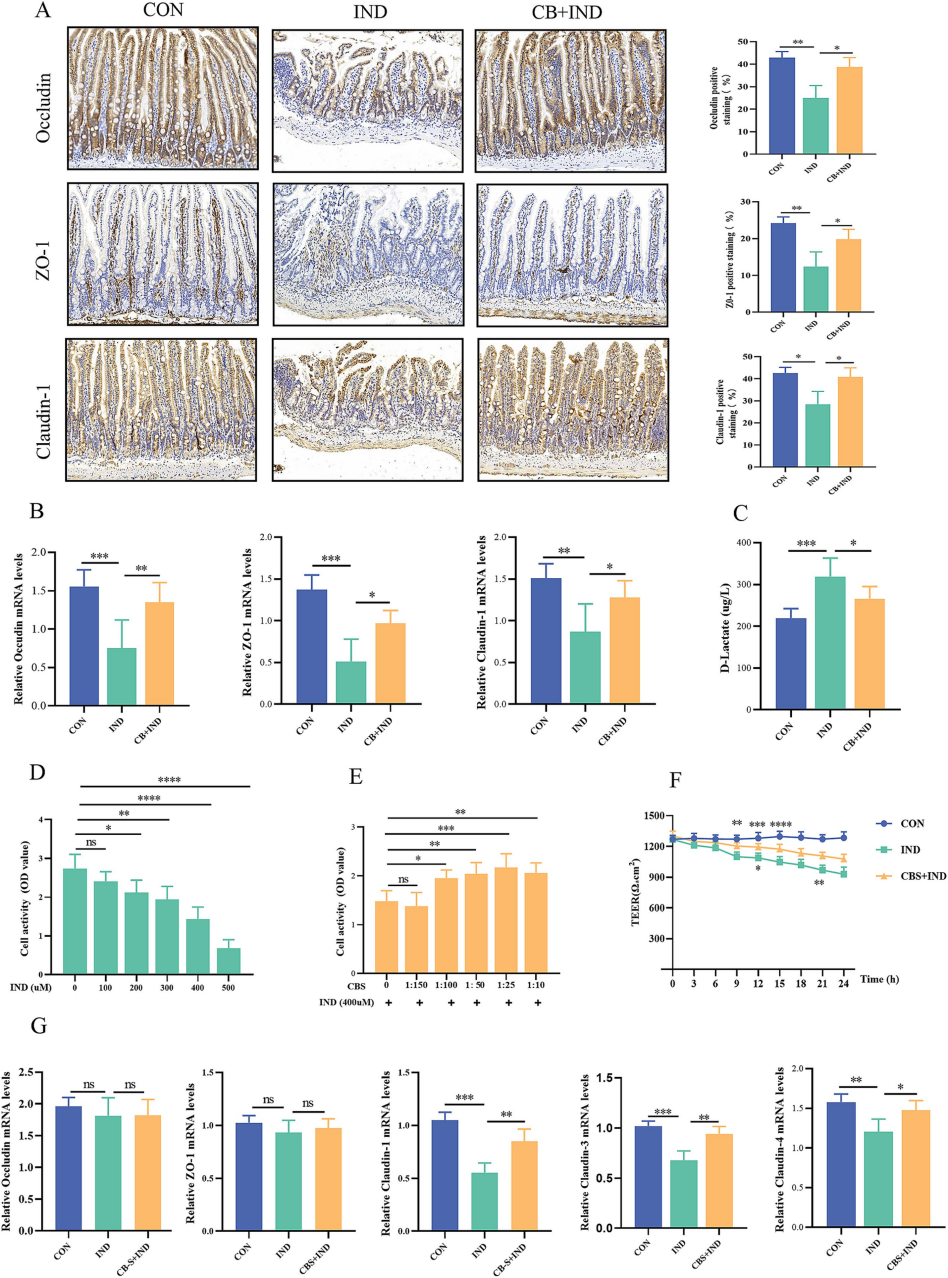
*Clostridium butyricum* (CB) protected the intestinal barrier function *in vivo* and *in vitro*. **(A)** Immunohistochemistry staining for occludin, ZO-1, and claudin-1 (scale bar = 200 μm, *n* = 3). **(B)** Relative mRNA expression of occludin, ZO-1, and claudin-1, as analyzed via qRT-PCR (*n* = 6). **(C)** Serum D-lactate levels measured by ELISA (*n* = 6). **(D)** Caco2 cells were stimulated with different concentrations of IND (0, 100, 200, 300, 400, and 500 μM) for 24 h, and the cell viability was measured by CCK-8 (*n* = 6). **(E)** Effect of different concentrations of CB supernatant (CBS; 0, 1:150, 1:100, 1:50, 1:25, and 1:10) on IND-induced viability changes (*n* = 6). **(F)** Relative mRNA expression of occludin, ZO-1, claudin-1, claudin-3, and claudin-4, as analyzed via qRT-PCR (*n* = 4). **(G)** Effect of CBS on IND-induced cell permeability changes, as measured by transepithelial electrical resistance (TEER, *n* = 4). Data were presented as mean ± standard deviation. ns, no significance; *p < 0.05, **p < 0.01, ***p < 0.001, and ****p < 0.0001.

NSAIDs can inhibit the oxidative phosphorylation of the mitochondria in enterocytes, leading to dysfunction of tight junction proteins and the mucosal barrier (Watanabe et al., 2011). The tight junction proteins (occludin, ZO-1, and claudin-1) of the small intestine are localized to the epithelial membrane and abundantly lost in NSAID enteropathy (Oncel and Basson, 2022). In the IND group, occludin, ZO-1, and claudin-1 protein-positive staining showed a clear decrease, with a scattered and discontinuous distribution. Meanwhile, in the CB + IND group, occludin, ZO-1, and claudin-1 protein-positive staining was observed to be increased (Figure 2A). Similarly, the occludin, ZO-1, and claudin-1 mRNA expression levels in the IND group were significantly lower compared to the CON group. In contrast, these levels in the CB + IND group showed an increase (Figure 2B).

Under normal physiological conditions, the serum level of D-lactate remained low, because this molecule is mainly derived from bacterial production and intestinal absorption instead of active synthesis of the human body. When the intestinal barrier is damaged, the serum concentration of D-lactate is increased due to its penetration into the mucosa and blood vessels (Sun et al., 2001). Therefore, D-lactate could be used as a biomarker to evaluate intestinal permeability. In the IND group, serum D-lactate levels were significantly higher than those in the CON group (*p* < 0.001). Conversely, in the CB + IND group, serum D-lactate levels were decreased compared to those in the IND group (*p* < 0.05, Figure 2C). These results indicated that CB could enhance the intestinal barrier and reduce intestinal permeability.

### 3.4 CBS protected cell barrier function

IND can reduce cell viability and damage cell barrier function (Omatsu et al., 2009). *In vitro*, Caco2 cells were treated with different concentrations of IND (0, 100, 200, 300, 400, and 500 μM) for 24 h. The results showed that the cell viability gradually decreased with the increase in IND concentration (Figure 2D). Advance 8 h action with different concentrations of CBS (0, 1:150, 1:100, 1:50, 1:25, and 1:10) and incubation with IND-induced Caco2 cells for 24 h resulted in enhanced cell viability (Figure 2E). The concentration of 400 μM IND and 1:25 CBS was selected as the experimental concentration to stimulate Caco2 cells. The expression of tight junction proteins was subsequently measured, and no significant difference was found in the expression of occludin and ZO-1 mRNA among the three groups. Nevertheless, the levels of claudin-1, claudin-3, and claudin-4 mRNA expression in the IND group notably decreased compared to those in the CON group (*p* < 0.001, *p* < 0.001, and *p* < 0.01). CBS treatment increased the three mRNA expression levels compared to IND group (*p* < 0.01, *p* < 0.01, and *p* < 0.05; Figure 2G). Meanwhile, the effect of CBS on cell permeability was indirectly evaluated by measuring the TEER value for 24 h. In the IND group, the TEER values gradually decreased with prolonged stimulation time and exhibited a notable downward trend, while CBS treatment ameliorated the reduction in IND-induced permeability (Figure 2F).

### 3.5 CB promoted proliferation and inhibited apoptosis in the small intestine

**Figure 3:**
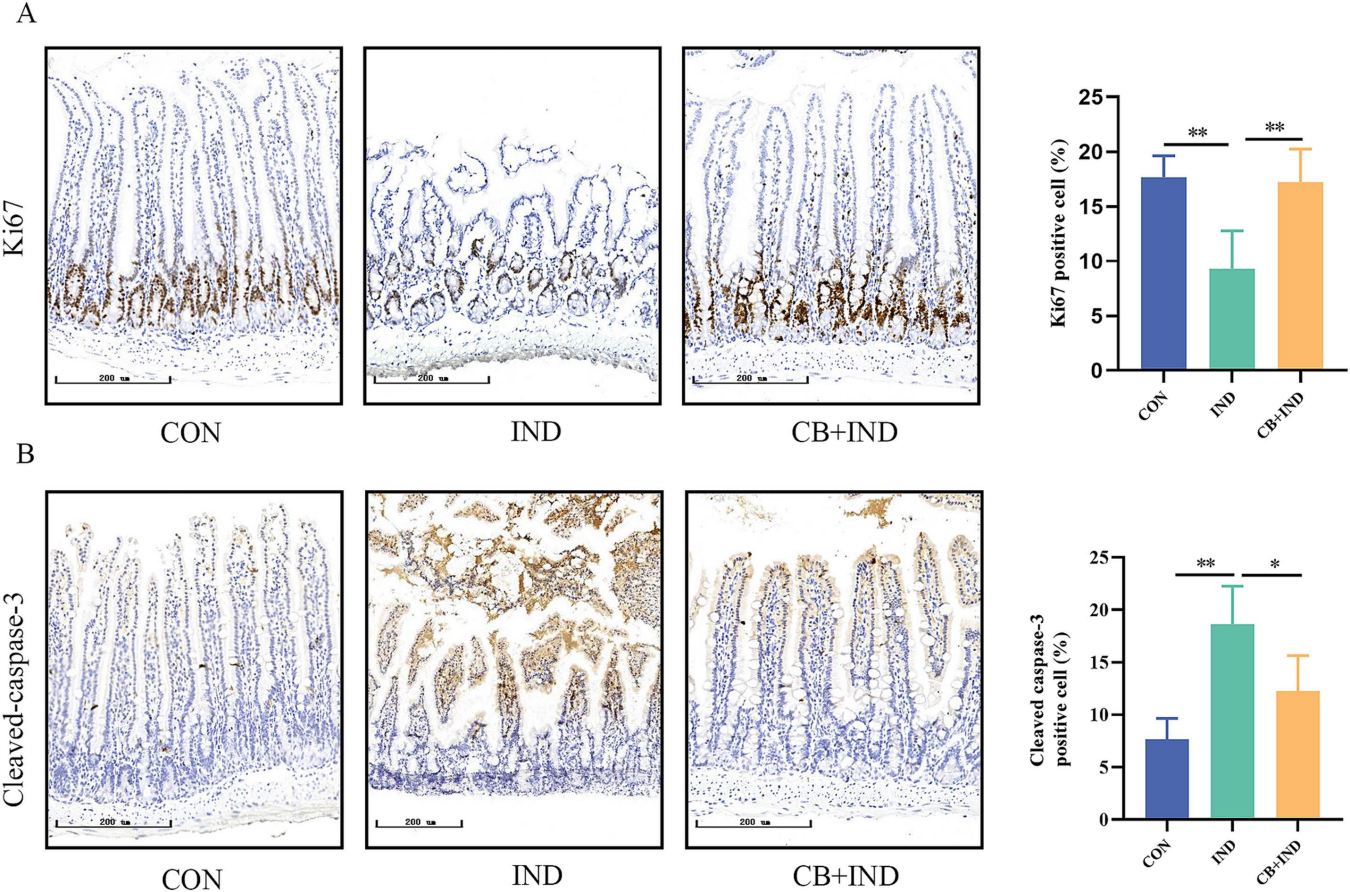
*Clostridium butyricum* (CB) promoted proliferation and inhibited apoptosis in the small intestine. **(A,B)** Immunohistochemical (IHC) staining for Ki-67 and cleaved caspase-3 (scale bar = 200 μm, *n* = 3). Data were presented as mean ± standard deviation. *p < 0.05 and **p < 0.01.

Proliferation, differentiation, and apoptosis are key factors in maintaining the integrity of the mucosal barrier in the small intestine (Ciccocioppo et al., 2002). Hence, proliferation in the small intestine was evaluated by IHC for Ki67. Compared to the CON group, the IND group exhibited a reduction in the number of Ki67-positive cells (9.31% ± 1.73% versus 17.65% ± 0.99%, *p* < 0.01), indicating that the impaired epithelial proliferation function was caused by IND. The number of Ki67-positive cells in the CB + IND group significantly increased compared to that in the IND group (17.21% ± 3.03%, *p* < 0.01, Figure 3A). Apoptosis in the small intestine was measured by IHC for cleaved caspase-3, a biochemical marker of apoptosis. The expression of cleaved caspase-3 was higher in the IND group compared to the CON group (18.62% ± 3.63% versus 7.67% ± 1.99%, *p* < 0.01). The CB + IND group showed decreased cleaved caspase-3 levels compared to the IND group (*p* < 0.05, Figure 3B). These results indicated that CB not only inhibited apoptosis but also promoted proliferation in the small intestine, thus maintaining the integrity of the mucosal barrier.

### 3.6 CB increased the number of goblet cells and MUC2 secretion

**Figure 4:**
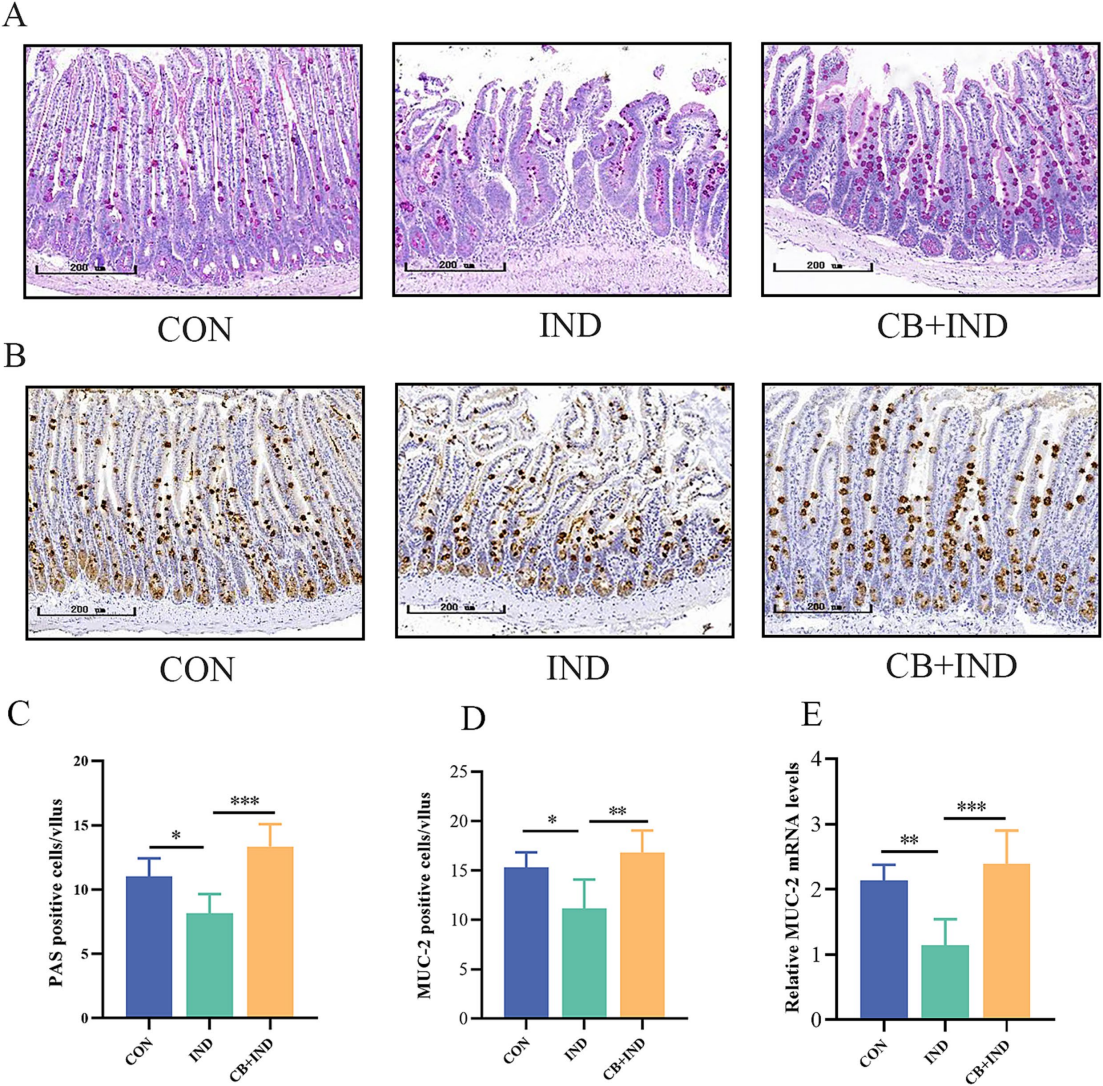
*Clostridium butyricum* (CB) increased the number of goblet cells and MUC2 secretion in the small intestine. **(A,C)** Goblet cells in the intestine were assessed by PAS staining, and the number of positive cells in each intestinal villus was calculated (scale bar = 200 μm, *n* = 6). **(B,D)** MUC2 expression in the intestine was detected by immunohistochemistry, and the number of positive cells in each intestinal villus was calculated (scale bar = 200 μm, *n* = 3). **(E)** Relative mRNA expression of MUC2 in intestinal tissues, as analyzed via qRT-PCR (*n* = 6). Data were presented as mean ± standard deviation. *p < 0.05, **p < 0.01, and ***p < 0.001.

Goblet cells are a subtype of intestinal secretory cells that mainly secrete mucin, a slimy glycoprotein maintaining the gut barrier. Among the different subtypes of secretory mucins, MUC2 is the major macromolecular component found in the small intestinal mucus layer. Therefore, the amount of goblet cells and MUC2 are potential biomarkers of the external barrier (Ma et al., 2018). In the present study, the amount of goblet cells on the small intestinal villi was measured by PAS staining. The IND group exhibited a reduction in PAS-positive cells compared to the CON group (*p* < 0.05). In contrast, the CB + IND group had markedly increased PAS-positive cells compared to the IND group (*p* < 0.001, Figures 4A,C). The expression of intestinal MUC2 was determined by IHC and qRT-PCR. The CB + IND group had a higher number of MUC2-positive cells in the small intestinal villi compared to the IND group (*p* < 0.01, Figures 4B,D). Additionally, the IND group exhibited reduced MUC2 mRNA expression compared to the CON group (*p* < 0.01), while the CB + IND group showed a significant increase in MUC2 mRNA (*p* < 0.001, Figure 4E). MUC5AC, a mucin commonly associated with inflamed tissue, is often upregulated in response to intestinal injury. The expression of intestinal MUC5AC was determined by IHC. The results showed that MUC5AC was almost not expressed in the three groups of small intestine tissues (Supplementary Figure S1).

The results revealed that NSAID administration led to a notable decrease in small intestinal goblet cells. Adding CB showed an impressive capacity to boost the goblet cell population in the small intestine. This indicates that CB supplementation could offset the harmful effects of NSAIDs on goblet cell numbers, potentially helping to maintain a healthy intestinal mucus barrier.

### 3.7 CB regulated the downstream pathway of Notch signaling

**Figure 5:**
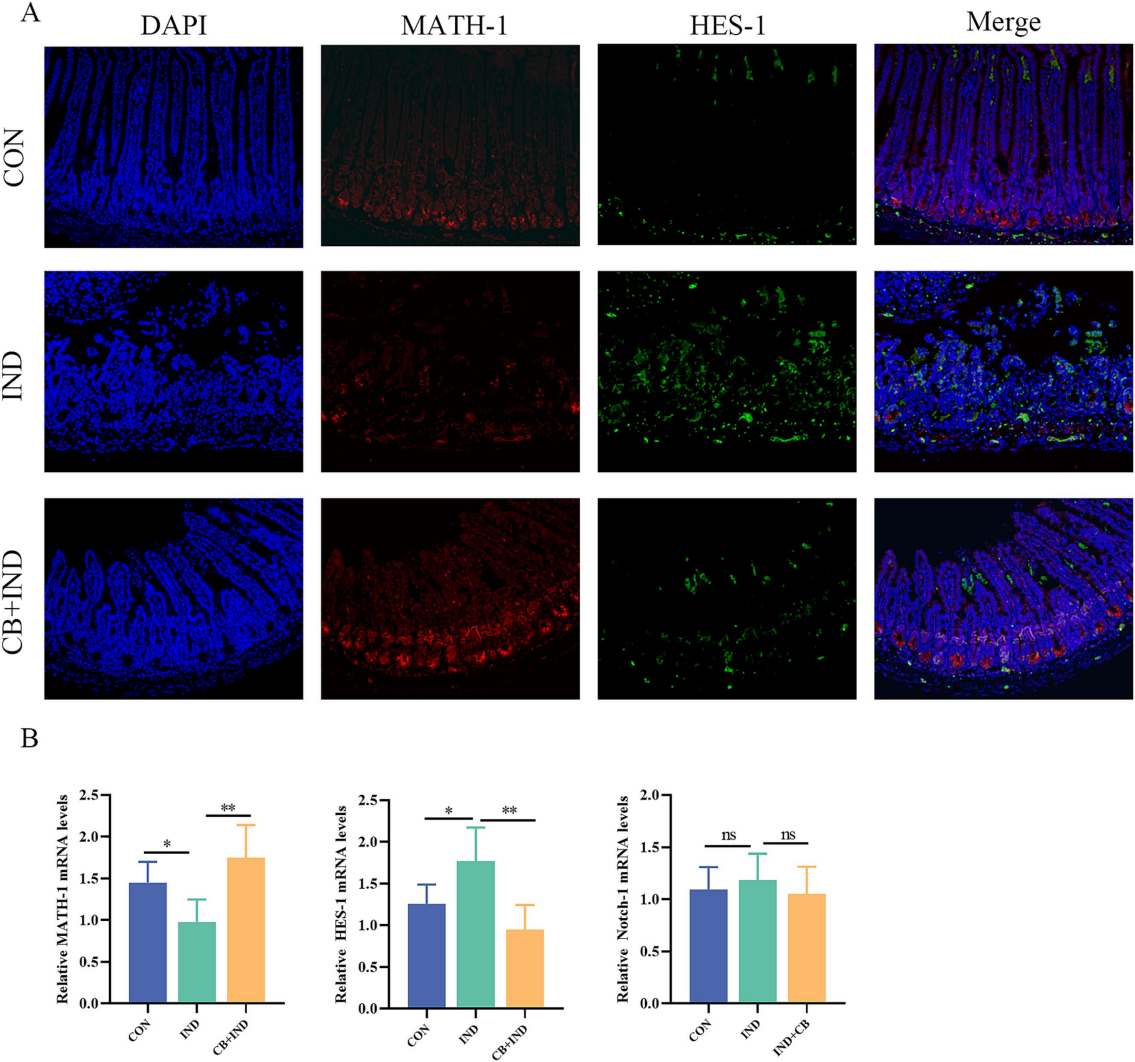
*Clostridium butyricum* (CB) regulated Notch signaling pathway in small intestine. **(A)** Immunofluorescence (IF) staining for HES-1 and MATH-1 (scale bar = 200 μm, *n* = 3). **(B)** Relative mRNA expression of MATH-1, HES-1, and Notch-1 in intestinal tissues, as analyzed via qRT-PCR (*n* = 6). Data were presented as mean ± standard deviation. *p < 0.05 and **p < 0.01.

In mammals, four types of Notch receptors (Notch1–4) were identified, of which Notch-1 was primarily distributed in the intestine (Yu and Canalis, 2020). To determine whether the effects of CB on goblet cell proliferation and differentiation depend on Notch signaling pathway, we investigated the changes in key factors HES1 and MATH1 first. The result showed that in the IND group, there was a significant increase in HES-1 protein expression and a decrease in MATH-1 protein expression. This suggests that the activation of the Notch signaling pathway occurred in the small intestinal tissues. In contrast, the CB + IND group exhibited a decrease in HES-1 protein expression and a noticeable increase in MATH-1 protein expression (Figure 5A). HES-1 transcript levels were found to increase in the IND group, but decreased upon treatment with CB. On the other hand, CB treatment upregulated the decreased expression of MATH-1 in the IND group (Figure 5B). There was no significant difference in the expression of the Notch-1 receptor among the three groups (Supplementary Figure S2), and similar results were observed in the Notch-1 mRNA expression (Figure 5B). These findings suggested that CB promoted the expression of MUC2 via suppressing the Notch signaling downstream pathway in small intestine.

### 3.8 CBS suppressed Notch signaling pathway in LS174T cells

**Figure 6:**
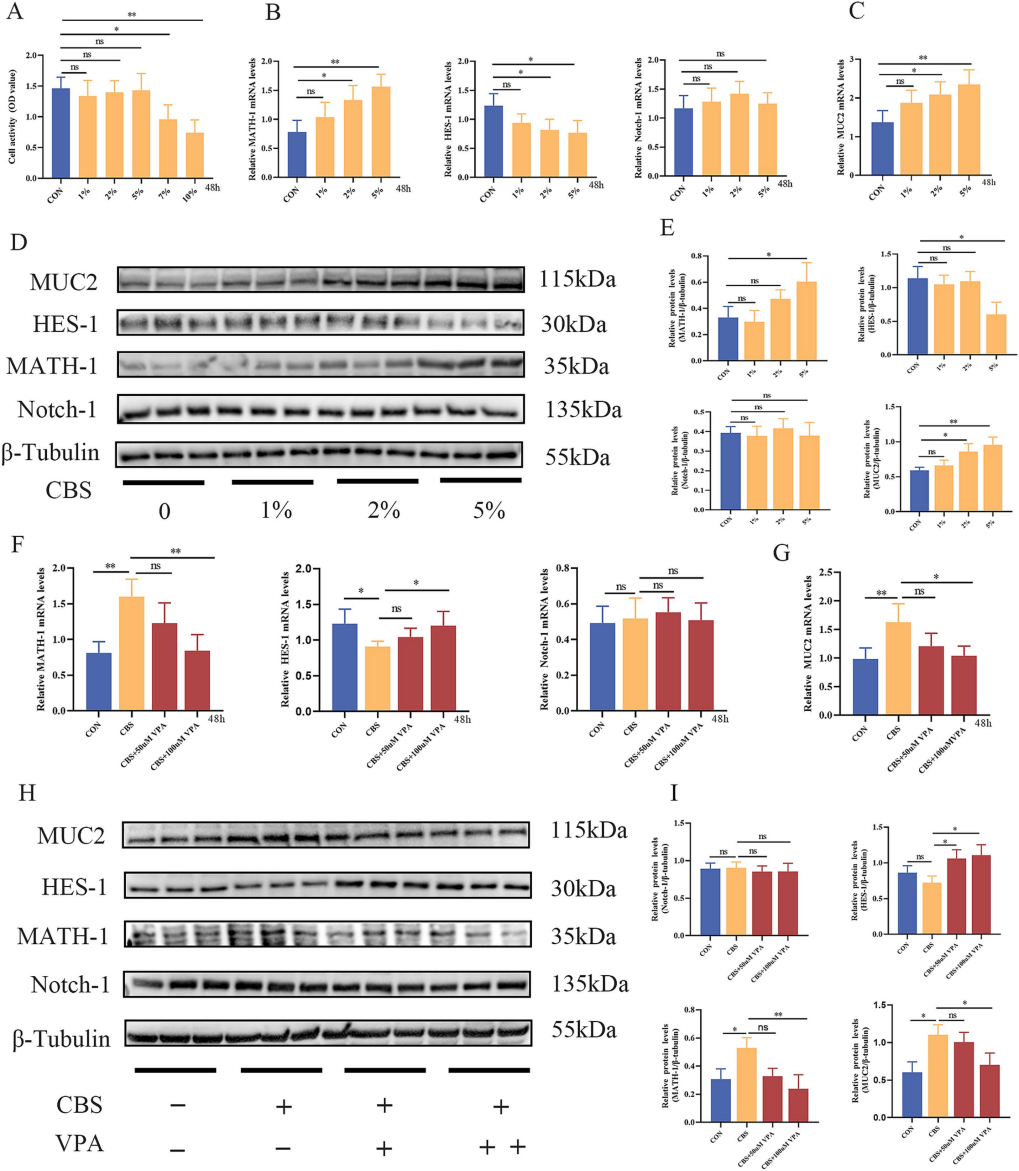
*Clostridium butyricum* supernatant (CBS) promoted the expression of MUC2 via suppression of Notch signaling pathway in LS174T cells. **(A)** Treatment with different concentrations of CBS (1, 2, 5, 7, and 10%) for 48 h. Cell viability was measured by CCK-8 (*n* = 6). **(B,C)** Relative expression of MATH-1, HES-1, Notch-1, and MUC2 mRNA induced by different concentrations of CBS (*n* = 4). **(D,E)** Western blotting and quantitative analysis of MATH-1, HES-1, Notch-1, and MUC2 proteins induced by different concentrations of CBS (*n* = 3). **(F,G)** Relative expression of MATH-1, HES-1, Notch-1, and MUC2 mRNA induced by CBS and different concentrations of valproic acid (VPA), an agonist of Notch signaling (*n* = 4). **(H,I)** Western blotting and quantitative analysis of MATH-1, HES-1, Notch-1, and MUC2 proteins induced by CBS and different concentrations of VPA (*n* = 3). Data were presented as mean ± standard deviation. *p < 0.05 and **p < 0.01.

LS174T cells exhibit characteristics of goblet cells and are commonly used to study intestinal diseases, dysfunction of the mucus layer, and signaling pathways related to goblet cells, such as the Notch signaling pathway (Kim et al., 2019). Next, our investigation will focus on determining whether the CB supernatant has a direct regulatory effect on goblet cells. Among the different concentrations of CBS tested, no significant impact on cell viability was observed at 1, 2, and 5% concentrations. However, at 7 and 10% concentrations of CBS, a notable decrease in cell viability was observed, indicating a suppressive effect on cell viability (Figure 6A). Thus, 1, 2, and 5% concentrations of CBS were selected to stimulate cells in order to primarily observe the impact of CBS on the Notch signaling pathway. The expression of HES-1 mRNA was inhibited by 2 and 5% CBS, while the expression levels of MATH-1 and MUC2 mRNA were increased (Figures 6B,C). The Western blotting results exhibited similar regulatory patterns in MUC2, HES-1, and MATH-1, indicating an obviously influence of the CBS on these factors. However, no significant effect on Notch-1 expression was observed (Figures 6D,E). Interestingly, when cells were stimulated with 50 or 100 μM VPA, an agonist of the Notch signaling pathway, it reversed the regulatory effect of CBS on the downstream molecules of the Notch pathway. This was evident from higher HES-1 expression and lower MATH-1 expression at the genetic and protein levels, compared to cells treated with CBS alone. Additionally, VPA also suppressed the expression of MUC2 induced by CBS. The expression of Notch-1 was not significantly affected by VPA (Figures 6F–I). These findings suggest that CBS may enhance the expression of MUC2 by inhibiting the activation of the Notch signaling pathway.

## 4 Discussion

Over the last several years, the upper gastrointestinal injury caused by NSAIDs has decreased, whereas the incidence of NSAID enteropathy has increased (Bjarnason et al., 2018), partially because PPIs can be used to treat NSAID-induced upper gastrointestinal injury, but they aggravate NSAID enteropathy (Zhang X. et al., 2023). Graham et al. (2005) reported that 71% of patients taking long-term NSAIDs had small intestinal damage. Therefore, managing NSAID enteropathy is an urgent task. In recent years, despite numerous studies on NSAID-related small intestinal injury, no promising treatment strategy has been identified. Thus, novel therapeutic approaches need to be explored.

Probiotics are commonly used in intestinal diseases for their effects in the regulation of microbiota dysbiosis, anti-inflammation, and barrier function enhancement. Probiotics, such as *Bifidobacterium breve* Bif195 and *Lacticaseibacillus casei*, can alleviate the clinical symptoms of patients suffering from NSAID enteropathy (Endo et al., 2011; Mortensen et al., 2019). However, the protection mechanisms have not been clearly defined. Not all probiotics have been found to effectively treat NSAID enteropathy. For instance, Kamil et al. (2007) found that *Lacticaseibacillus rhamnosus* GG (LGG) aggravated the small intestinal damage induced by IND. However, another study showed that LGG supernatant contributed to the production of intestinal mucin and regulated intestinal microflora (Gu et al., 2022), implying the multiple effects of probiotics on human health. Currently, CB has been applied to intestinal diseases, such as IBD and IBS (Yasueda et al., 2016; Sun et al., 2018). The precise mechanisms of the beneficial effects of CB remain unclear, but in addition to its direct effect on the intestinal microbiota, the intestinal barrier-enhancing and anti-inflammatory properties of CB metabolites seem to contribute to its efficacy against NSAID-induced enteropathy. Therefore, we used CB for the mice and CBS (containing metabolites of CB) for the *in vitro* models. In the present study, the protective effect and potential mechanisms of CB on IND-induced enteropathy were investigated. The results showed that CB and its secretions may improve IND-induced enteropathy by promoting the secretion of MUC2 via suppressing the Notch signaling pathway (Supplementary Figure S3). Meanwhile, CB was found to suppress the inflammatory response, promote epithelial cell proliferation, and inhibit apoptosis. In addition, CB and CBS enhanced the expression of tight junction proteins and decreased cell permeability.

NSAIDs are a class of drugs used to treat inflammatory diseases, and they inhibit the release of pro-inflammatory factors. When intestinal barrier function is damaged due to NSAID, luminal lipopolysaccharide (LPS) translocation leads to immune cell activation, exceeding the anti-inflammatory effect of NSAID, thereby leading to an inflammatory response (Chen et al., 2021). Neutrophil infiltration plays a crucial role in NSAID enteropathy (Otani et al., 2017). The number of Ly6G-positive cells and fecal calprotectin levels are markers of neutrophil infiltration (Lee et al., 2013; Chamoun-Emanuelli et al., 2019). In the present work, IND increased the Ly6G-positive cells in the small intestinal mucosa, increased the fecal calprotectin levels, and shortened small intestine length. By contrast, CB treatment improved these indicators. TNF-α and IL-1β are closely associated with the inflammatory response, and their increase could lead to a reduction in intestinal barrier function and aggravate inflammatory response (Friedrich et al., 2019). IL-10 not only alleviates the inflammatory response, but also regulates the epithelial integrity of the small intestine (Morhardt et al., 2019). In the present study, CB decreased the expression of TNF-α and IL-1β mRNA and promoted that of IL-10 mRNA. Besides, IL-10 could contribute to the correct folding of MUC2 and promote the maturation of goblet cells by repressing Notch signal (Hasnain et al., 2013; Deng et al., 2020), thus providing support for follow-up research to some extent. In the experimental design, we included 0.5% CMC in the control group. However, CMC has been shown to alter the gut microbiota in mice and promote inflammation (Chassaing et al., 2015). CMC is a common water-soluble cellulose derivative with various uses in animal experiments, including as a solvent or suspending agent for drugs or compounds, and as a vehicle for control groups. In our study, CMC was used to prepare suspensions of poorly soluble drug indomethacin, ensuring its uniform dispersion in solution in the treatment groups. Since the treatment groups received indomethacin suspended in 0.5% CMC, it was essential to include the same concentration of CMC in the control group to maintain consistency and eliminate any potential confounding effects arising from the vehicle itself. The CMC concentration (0.5%) and duration (1 week) used in our study were lower than the levels typically associated with significant gut microbiota alterations or inflammatory responses in mice. Furthermore, our study suggest that 0.5% CMC in the control group does not induce significant physiological changes in the short-term experimental duration.

Intestinal integrity is the basic guarantee of intestinal function. Intestinal integrity is maintained by several vital lines of defense, including mechanical barrier, chemical barrier, immunological barrier, and biological barrier. The mechanical barrier is dependent on tight junction proteins, which are the determinants of intestinal barrier function. They are primarily constituted by occludin, claudins and ZO-1 proteins, which prevent damage to the mucosa by hazardous substances and preserve the integrity of the epithelium. When tight junction proteins were damaged, the permeability between intestinal epithelial cells was increased (Schneeberger and Lynch, 1992; Xia et al., 2019). According to Shi et al. (2014), rats administered with IND had reduced expression of occludin and ZO-1 in intestinal tissue. Besides, MKN-28 cells exposure to IND decreased the expression of occludin without changing that of ZO-1 and claudin-1 (Thakre-Nighot and Blikslager, 2016). Consistent with these, our results showed that IND reduced the expression of occludin, claudin-1 and ZO-1, and increased intestinal permeability, which were improved by CB administration. Moreover, our study found that IND decreased the mRNA expression of claudin-1, claudin-3, and claudin-4 without affecting that of ZO-1 and occludin in Caco2 cells, and CBS alleviated the negative effects of IND on tight junction proteins and reduced Caco2 cell permeability. These results demonstrated that CB protected against NSAID enteropathy via enhancing the intestinal barrier function.

Normal tissue homeostasis depends on the balance between epithelial cell apoptosis and proliferation (Jiang et al., 2022). Previous studies have shown that cytokines, such as TNF-α and IL-1β, can drive intestinal epithelial apoptosis (Cai et al., 2023). An increase in intestinal epithelial cell apoptosis could cause damage to the intestinal barrier function and consequently lead to intestinal inflammation (Camilleri, 2019). As is well known, IND promotes apoptosis of intestinal epithelial cells (Omatsu et al., 2009). The present study demonstrated that IND suppressed the proliferation and promoted the apoptosis of intestinal epithelial cells, which could be improved following the administration of CB.

The mucus layer is an essential component of the intestinal barrier, which provides defense of the epithelial cells from pathogens (Capaldo et al., 2017). Goblet cells secrete MUC2, which is the fundamental backbone of the mucus layer, and IBD is accompanied by depletion of goblet cells and decreased expression of MUC2 (Jiang et al., 2022). MUC2^−/−^ mice may develop IBD spontaneously, possibly due to direct contact between intestinal microorganisms and epithelial cells (Hansson and Johansson, 2010). The results of the present study showed that CB could alleviate IND-induced depletion of goblet cells and promote the expression of MUC2. MUC2 has a large molecular weight of about 540 kDa, and its molecular weight can exceed 2 MDa after glycosylation. Commercially available MUC2 antibodies predict a molecular weight of 540 kDa for MUC2. However, the actual molecular weight observed in Western blot is less than 540 kDa, mostly around 100–200 kDa. The observed molecular weight of MUC2 is influenced by both its predominant O-glycosylation profile (in contrast to N-glycosylation) and the glycosylation capacity of the chosen expression system (Gallego et al., 2023). For example, the use of CHO cells—a widely adopted platform with inherent limitations in O-glycosylation efficiency—may lead to simplified glycan structures, thereby contributing to apparent molecular weight discrepancies. In addition, the observed molecular weight of the protein may vary from the predicted molecular weight due to post translational modifications, post translation cleavages, relative charges, and other experimental factors. It is noteworthy that LS174T cells, while widely used for mucin studies, exhibit cancer-associated alterations in glycosylation pathways. These inherent limitations may result in truncated or atypical glycan structures on recombinant MUC2 compared to native mucins secreted in healthy human tissues (van Klinken et al., 1996). MUC5AC, a secreted gel-forming mucin, has garnered increasing attention for its role in intestinal inflammation. MUC5AC is usually expressed in the stomach, trachea, and bronchus, and is typically absent in the healthy colon, but is markedly upregulated in ulcerative colitis (UC) and experimental colitis (Olli et al., 2020; Shaoul et al., 2004). This induction likely represents an adaptive response of goblet cells to inflammatory damage, compensating for insufficient MUC2 function or mucus layer disruption (Olli et al., 2020; Grondin et al., 2020). For instance, *MUC5AC*^−/−^ mice exhibit exacerbated tissue damage, heightened inflammation, and increased bacterial penetration through the mucus layer in DSS colitis, underscoring MUC5AC’s protective role (Olli et al., 2020). MUC5AC collaborates with MUC2 to form a gel-like mucus layer, limiting direct bacterial-epithelial contact (Olli et al., 2020), and binds pathogens (e.g., *Citrobacter rodentium*) for clearance via mucus flow, reducing colonization (Lindén et al., 2008). In UC patients, MUC5AC co-expresses with gastric trefoil factor 1 (TFF1) in goblet cells, potentially promoting epithelial migration and repair (Shaoul et al., 2004). Previous studies in mice have also shown a protective role for MUC5AC (murine) in helminth infection, suggesting that MUC5AC expression in the gastrointestinal tract is a tissue-protective response (Hasnain et al., 2011). However, our study showed that MUC5AC was almost not expressed in the three groups of small intestine tissues.

Notch signaling plays a key role in regulating differentiation, proliferation, and apoptosis in various tissues. The Notch signaling pathway is composed of Notch receptors, Notch ligands, and DNA-binding proteins, in which Notch-1 is primarily distributed in the mammalian intestine (Zhou et al., 2022). When the Notch pathway is activated, Notch-1’s intracellular structural domain (NICD) binds to RBP-Jk and activates the expression of transcription factor HES-1; then, HES-1 binds to the 5′ promoter domain of MATH-1 directly and inhibits the expression of the transcription factor MATH-1, which consequently results in limiting the differentiation of intestinal stem cells to goblet cells and leading to mucus barrier dysfunction (Shinoda et al., 2010). Park et al. (2006) discovered that MATH-1 promoted MUC2 expression via binding to MUC2 promoter. Our results showed that CB promoted the secretion of MUC2 by suppressing Notch signaling. Meanwhile, CBS promoted MUC2 secretion by suppressing Notch signaling in LS174T cells. The promotion of MUC2 facilitated by CBS could be impeded following the utilization of VPA, an agonist of Notch signaling, further suggesting that CBS could enhance MUC2 expression by inhibiting Notch signaling.

However, there are still several limitations in this study. The effects of CB on the small intestinal microbiota need to be investigated. In addition, the study could not definitively prove that CB promotes goblet cell differentiation and maturation by inhibiting Notch signaling. Further organoid experiments are necessary to confirm this hypothesis. Furthermore, studying the bioactive components (such as proteins, short-chain fatty acids, tryptophan metabolites, polysaccharides, and small peptides) of CB that ameliorate NSAID enteropathy is a crucial matter and is the main focus of our upcoming research.

## 5 Conclusion

Our study demonstrated that CB could ameliorate IND-induced enteropathy by promoting the secretion of MUC2 by goblet cells through inhibiting the Notch signaling pathway. These findings highlight the potential of CB as a supplementary intervention for the treatment of NSAID enteropathy. Furthermore, our results underscore the importance of further investigating and developing novel therapeutic approaches in this field.

## Data Availability

The raw data supporting the conclusions of this article will be made available by the authors, without undue reservation.
