# Supplementary material for: Clostridium butyricum ameliorates indomethacin-induced enteropathy by promoting MUC2 secretion via suppressing the Notch pathway
Source: Front Microbiol. 2025 Mar 19;16:1509876. doi: 10.3389/fmicb.2025.1509876 (PMC11961966; doi:10.3389/fmicb.2025.1509876)
Supplement: Supplementary file 1 [file Data_Sheet_1.DOCX]

Supplementary Material

# Supplementary Figures and Tables

## Supplementary Figures


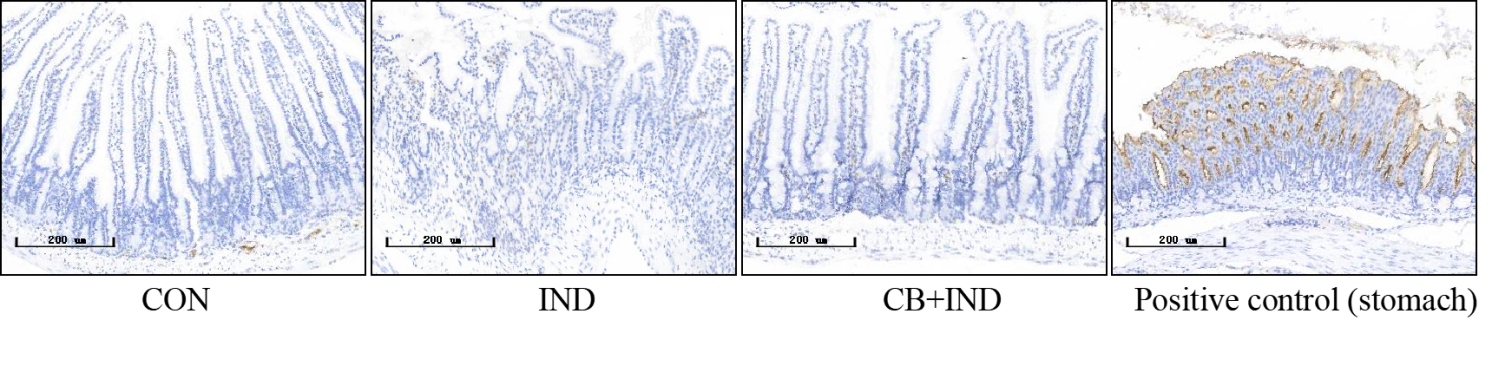
**Fig. S1** Effect of *Clostridium butyricum* (CB) on expression of MUC5AC in small intestine. Immumohistochemical staining for MUC5AC (scale bar = 200 μm, n = 6).


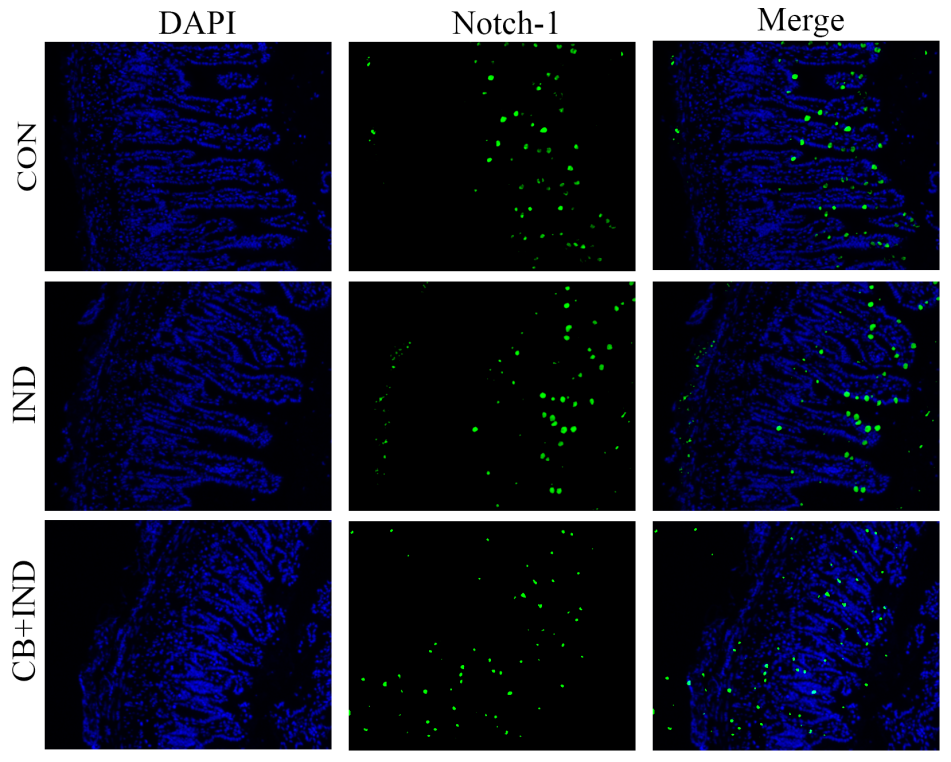


**Fig. S2** *Clostridium butyricum* (CB) regulates Notch signaling pathway in small intestine. Immunofluorescent staining for Notch-1 (scale bar = 250 μm, n = 3).


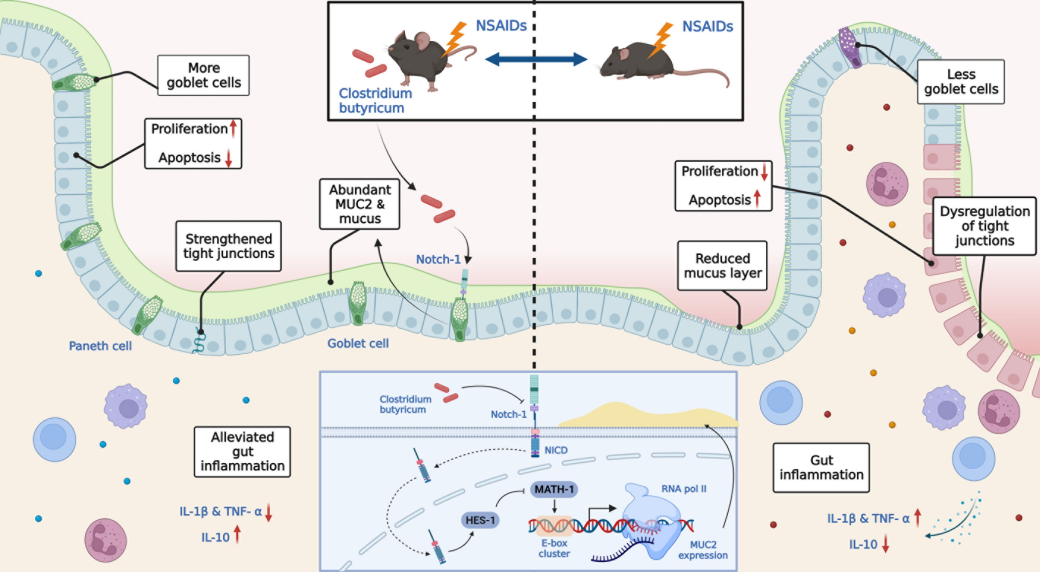


**Fig. S3** Summary of the protective effect of *Clostridium butyricum* on indomethacin-induced enteropathy and its effect on Notch signaling pathway.

## Supplementary Tables

**Table S1** Primers used in this study.

| Gene | Forward sequence (5 ′→ 3 ′ ) | Reverse sequence (5 ′→ 3 ′ ) |
| --- | --- | --- |
| Mice -GAPDH | TGTGTCCGTCGTGGATCTGA | CCTGCTTCACCACCTTCTTGA |
| Mice - IL-1β | GCAACTGTTCCTGAACTCAACT | ATCTTTTGGGGTCCGTCAACT |
| Mice - TNF-α | CTTCTGTCTACTGAACTTCGGG | CAGGCTTGTCACTCGAATTTTG |
| Mice -IL-10 | TGGACAACATACTGCTAACCG | GGATCATTTCCGATAAGGCT |
| Mice - ZO-1 | TGCAATTCCAAATCCAAACC | AGAGACAAGATGTCCGCCAG |
| Mice - Claudin-1 | AGACCTGGATTTGCATCTTGGTG | TGCAACATAGGCAGGACAAGAGTTA |
| Mice -Occludin | TGAAAGTCCACCTCCTTACAGA | CCGGATAAAAAGAGTACGCTGG |
| Mice -MUC2 | ACAAAAACCCCAGCAACAAG | GAAGTCGGGACAGGTGATGT |
| Mice - Notch1 | ACATCCGTGGCTCCATTGTCTA | TCTTGTAAGGAATATTGAGGCTGC |
| Mice - MATH-1 | AAAGGAGGCTGGCAGCAA | TGGTTCAGCCCGTGCAT |
| Mice - HES-1 | AAAATTCCTCCTCCCCGGTG | TTTGGTTTGTCCGGTGTCG |
| Human- GAPDH | TCGGAGTCAACGGATTTGGT | TTCCCGTTCTCAGCCTTGAC |
| Human -Occludin | ATGGCAAAGTGAATGACAAGCGG | CTGTAACGAGGCTGCCTGAAGT |
| Human- ZO-1 | TGCAATTCCAAATCCAAACC | AGAGACAAGATGTCCGCCAG |
| Human- Claudin-1 | TTAGTGGCCACAGCATGGTA | GAAGGTGTTGGCTTGGGATA |
| Human-Claudin-3 | CCAACTGGGTACAAGACGAG | TCTTGGTGGGTGCATACTTG |
| Human- Claudin-4 | GGAGGGCCTCTGGATGAACT | GATGCTGATGACCATAAGGGC |
| Human-Notch1 | GGTGAACTGCTCTGAGGAGATC | GGATTGCAGTCGTCCACGTTGA |
| Human-HES-1 | TCAACACGACACCGGATAAAC | GCCGCGAGCTATCTTTCTTCA |
| Human- MATH-1 | CCTTCCAGCAAACAGGTGAAT | TTGTTGAACGACGGGATAACAT |
| Human- MUC2 | ACCCGCACTATGTCACCTTC | GGACAGGACACCTTGTCGTT |
