# Supplementary figures and images for: Clostridium butyricum ameliorates indomethacin-induced enteropathy by promoting MUC2 secretion via suppressing the Notch pathway
Source: Front Microbiol. 2025 Mar 19;16:1509876. doi: 10.3389/fmicb.2025.1509876 (PMC11961966; doi:10.3389/fmicb.2025.1509876)

*MUC2*

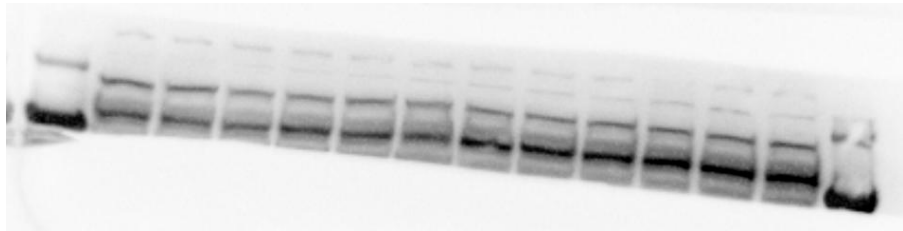

*HES-1*

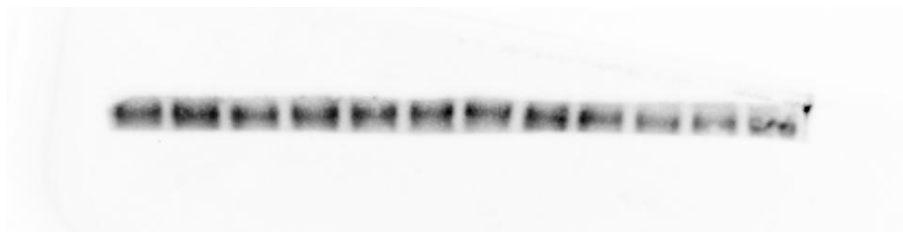

*MATH-1*

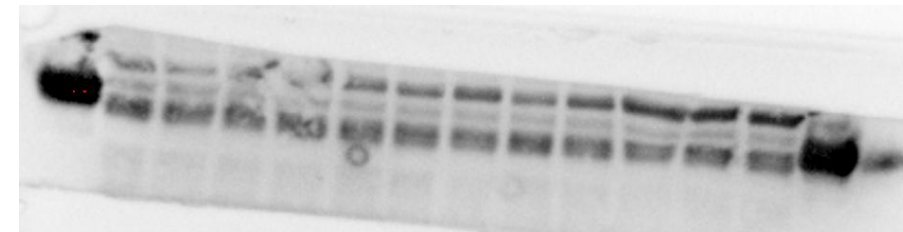

*Notch-1*

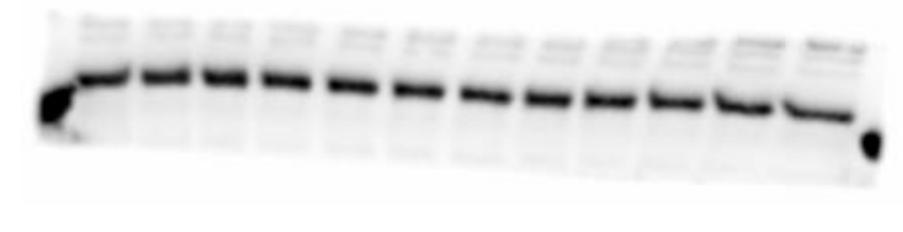

$\beta$ -Tubulin

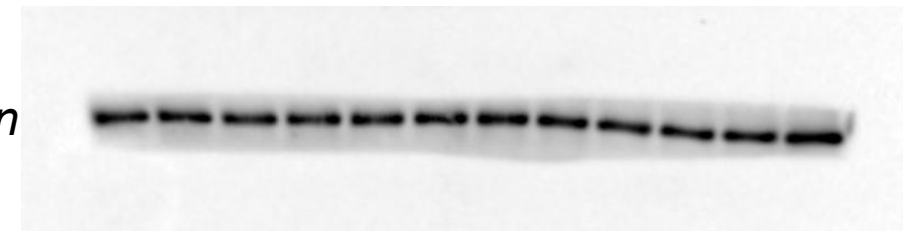

CBS

0      1%      2%      5%

*MUC2*

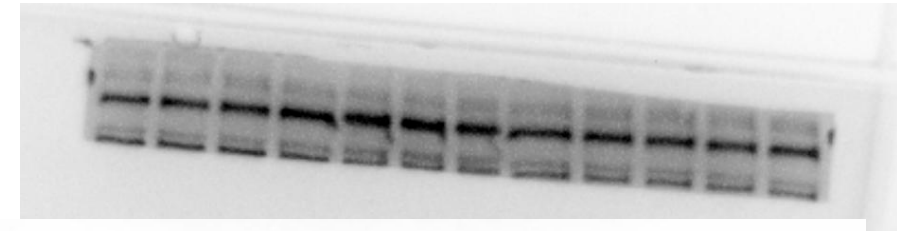

*HES-1*

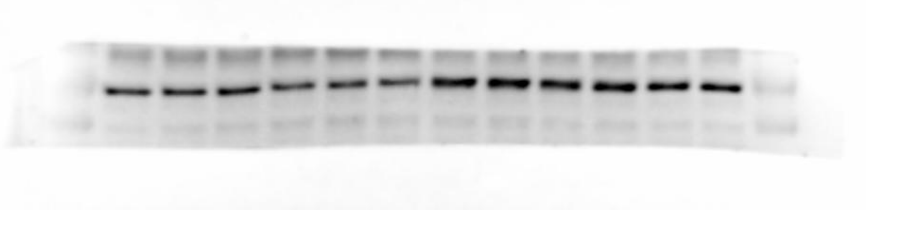

*MATH-1*

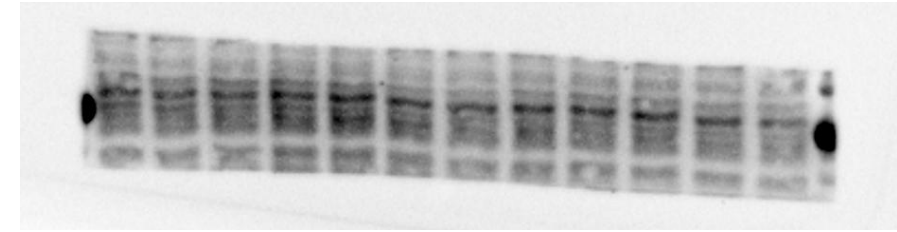

*Notch-1*

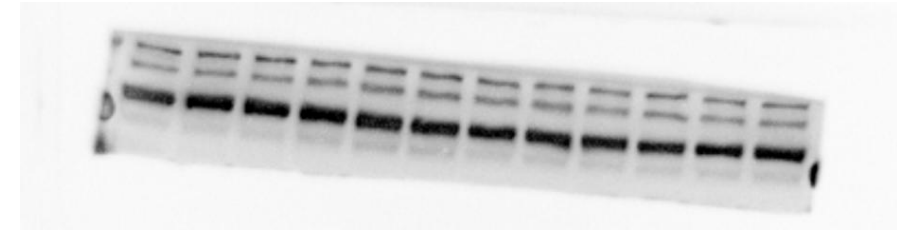

$\beta$ -Tubulin

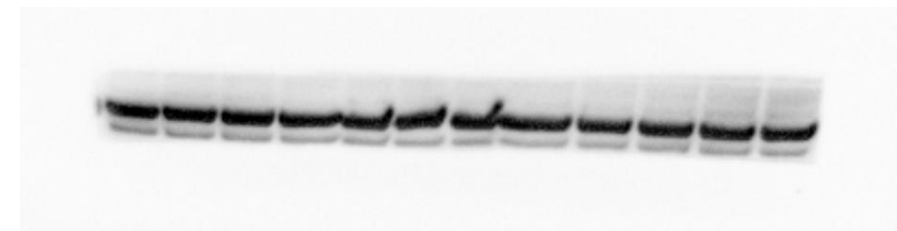

CBS

-      +      +      +

VPA

-      -      +      ++

Supplement: Supplementary file 2 [file Data_Sheet_2.PDF]
